# Health hazards to wild birds and risk factors associated with anthropogenic food provisioning

**DOI:** 10.1098/rstb.2017.0091

**Published:** 2018-03-12

**Authors:** Becki Lawson, Robert A. Robinson, Mike P. Toms, Kate Risely, Susan MacDonald, Andrew A. Cunningham

**Affiliations:** 1 Institute of Zoology, Zoological Society of London, Regent's Park, London NW1 4RY, UK; 2 British Trust for Ornithology, The Nunnery, Thetford, Norfolk IP24 2PU, UK; 3 Fera Science Ltd, National Agri-Food Innovation Campus, Sand Hutton, York YO41 1LZ, UK

**Keywords:** garden bird feeding, epidemiology, finch trichomonosis, Paridae pox, passerine salmonellosis, mycotoxin

## Abstract

Provision of supplementary food for wild birds at garden feeding stations is a common, large-scale and year-round practice in multiple countries including Great Britain (GB). While these additional dietary resources can benefit wildlife, there is a concomitant risk of disease transmission, particularly when birds repeatedly congregate in the same place at high densities and through interactions of species that would not normally associate in close proximity. Citizen science schemes recording garden birds are popular and can integrate disease surveillance with population monitoring, offering a unique opportunity to explore inter-relationships between supplementary feeding, disease epidemiology and population dynamics. Here, we present findings from a national surveillance programme in GB and note the dynamism of endemic and emerging diseases over a 25-year period, focusing on protozoal (finch trichomonosis), viral (Paridae pox) and bacterial (passerine salmonellosis) diseases with contrasting modes of transmission. We also examine the occurrence of mycotoxin contamination of food residues in bird feeders, which present both a direct and indirect (though immunosuppression) risk to wild bird health. Our results inform evidence-based mitigation strategies to minimize anthropogenically mediated health hazards, while maintaining the benefits of providing supplementary food for wild birds.

This article is part of the theme issue ‘Anthropogenic resource subsidies and host–parasite dynamics in wildlife’.

## 1. Introduction

### (a) Garden bird feeding in Great Britain

With habitat loss, degradation and progressive urbanization, there is increased focus on the value that domestic gardens provide for wild birds. Supplementary feeding of garden birds is practised by millions of people across Europe, North America and Australasia [1]. Wild bird feeding is postulated to be one of the most common forms of human–wildlife interaction in the Western world [2] and an estimated 48% of households in Great Britain (GB) provide supplementary food [3]. Since the 1970s, there has been a shift from winter-only to year-round feeding, supported by the argument that nutritional demands vary across the year and are not limited to periods of harsh weather. Concurrently, diversification in commercially available products, notably seed mixes, suet-based products and insectivorous diets, has occurred. This has been coupled with an increased variety of food presentation, from table and ground feeding options to numerous designs and sizes of suspended feeders.

Supplementary feeding may affect wild birds in many ways, including changes to body condition, reproductive success, survival, community structure and migration behaviour [4–7]. In GB, supplementary feeding has been linked to increases in population size of wild bird species making use of this resource [8]. It is also important to recognize that there is a human well-being perspective to the feeding of wild birds. Contact with wildlife in peri-domestic habitats offers an opportunity to address the growing disconnect with nature that has accompanied progressive urbanization. Feeding birds has been shown to promote human health and well-being and may enhance public interest in wildlife welfare and conservation [9,10], although it risks assuaging guilt over wider detrimental environmental change and habitat losses through the act of ‘doing good’ in one's immediate vicinity. While supplementary feeding has the potential to offset some losses resulting from reduced natural food supplies [11], the benefits largely seem to accrue to already urban-adapted species that frequently use feeding stations [12].

There are risks associated with feeding birds, which include the possibility that wild birds may become reliant on artificial food sources, or be subject to increased predation at feeding stations (e.g. [13,14]). Supplementary feeding also may increase opportunities for pathogen exposure and transmission, with risks associated with (i) congregation at high density for prolonged periods of time and repeatedly over long periods; (ii) opportunities for interspecific mixing unlikely to occur in natural habitats; and (iii) poor hygiene levels leading to pathogen contamination of the feeding station [15,16]. Also, supplementary food may pose a risk if it is of poor nutritional value or contaminated with toxins that influence host condition or immunity [16,17]. Finally, crowding and competition at feeding stations may lead to stress and secondary immunosuppression, with resources partitioned through dominance hierarchies [16,18]. Furthermore, the zoonotic potential of some wild bird pathogens is well known, and the close human–wildlife proximity at feeding stations may increase the risks to public health [19].

### (b) Citizen science for wildlife disease surveillance

Citizen science offers a cost-effective means to undertake large-scale, year-round, longitudinal disease surveillance in conjunction with the monitoring of wildlife populations, their distributions and abundances [20]. This approach lends itself to monitoring species, such as songbirds, that use peri-domestic habitats and are positively perceived by the public [21]. In GB, national wild bird disease surveillance has been achieved over a 25-year period through public reporting of observed morbidity and mortality. The methods used have evolved over this period in several ways, most notably involving a shift from opportunistic reports only (1992–2004) to an integrated system of independent opportunistic and systematic surveillance approaches (2005–present) [22], which has been greatly facilitated by expansion of an already existing national citizen science scheme [23].

Briefly, volunteers in the British Trust for Ornithology's (BTO) Garden BirdWatch (GBW) scheme submit weekly reports throughout the calendar year, providing measures of occurrence and abundance of wild birds using their gardens. Expansion of this scheme to include the recording of observation of disease now provides a structured and systematic dataset that can be used to control for the spatial and temporal biases seen in opportunistic reporting approaches. Post-mortem examinations are conducted from a subset of incidents, including those reported by both GBW participants and opportunistic recorders. Standardized examination protocols, supported by ancillary diagnostic tests, are conducted, and set case and incident definitions are employed (see the electronic supplementary material). A collaborative approach, bringing together veterinary diagnostic laboratories, conservation and animal welfare organizations, government, the wild bird care industry and academia, underpinned by public contributions, has been adopted to maximize awareness and impact across invested communities.

### (c) Scope of review

Appraisal of disease transmission risk associated with supplementary feeding relies on a combination of observation and experimental data. There are a few large-scale and long-term field datasets for disease surveillance of wild birds that frequent garden feeding stations. For small passerines in North America, the investigation of the spread of house finch conjunctivitis is perhaps the best-studied example. This has combined examination of field data, to provide information about spatio-temporal disease spread and house finch (*Haemorhous mexicanus*) population declines [24], with experimental studies to elucidate factors (e.g. behaviour) influencing *Mycoplasma gallisepticum* transmission (e.g. [25,26]). This bacterial infection results in externally visible, characteristic signs of conjunctivitis; therefore, syndromic surveillance through public reporting of affected bird sightings can be used as a reliable proxy for disease occurrence. However, for most types of disease, clinical signs are rarely this identifiable or specific, so alternative approaches are required.

By combining large-scale surveillance with post-mortem examinations, we can differentiate between multiple diseases that result in non-specific clinical signs (e.g. lethargy and fluffed-up plumage) that could not be diagnosed through observation alone. We focus on three of the best-characterized and most frequently diagnosed infectious diseases, with contrasting modes of transmission, caused by a protozoal, a viral and a bacterial pathogen, each of which has been known to occur over the duration of the study period (1992–2016) but for which the epidemiology, prevalence and impact have changed markedly over the past decade.

## 2. Finch trichomonosis

*Trichomonas gallinae* is a protozoan parasite that causes avian trichomonosis, a disease long known to affect columbiforms (pigeons and doves) and birds of prey [27]. The disease is generally characterized by necrotic upper alimentary tract lesions, which often interfere with the ability to swallow. Parasite transmission occurs via fresh saliva during conspecific feeding in courtship or when rearing young, or at shared food and water sources. Birds of prey are exposed to the parasite when they predate or scavenge infected avian hosts. The parasite has short-term environmental persistence and is killed by desiccation.

### (a) Pattern of occurrence of finch trichomonosis

Avian trichomonosis has long been documented sporadically in columbiforms and birds of prey in GB, its widespread distribution being consistent with an endemic disease [28]. Trichomonosis emerged in finch species in GB in spring 2005, with a presentation of necrotic ingluvitis [29]. Since its detection, the disease has been most frequently diagnosed in greenfinch *Chloris chloris* and chaffinch *Fringilla coelebs*; however, we have also diagnosed fatal trichomonosis in most of the gregarious, seed-eating species that visit garden feeding stations. Epidemic mortality occurs each year with a seasonal peak in late summer (August–September), although incidents continue year-round. Fatalities due to the disease have been recorded infrequently in other common garden birds, probably from spillover at sites where there are high levels of contamination of shared food and water sources and of the ground below bird feeders. Since its emergence, finch trichomonosis has been the most common infectious disease diagnosed for finch species (family Fringillidae), accounting for 87% (372/426) of finch infectious disease diagnoses over the period 2010–2016 inclusive (i.e. since the previous large-scale study on this disease [30]).

While multiple *T. gallinae* strains are known to infect pigeons and doves in GB, a single clonal A1 strain is responsible for the trichomonosis epidemic in European finches [31,32]. Although it is possible that this is a recently emerged, highly virulent strain with increased host range, we hypothesize that the epidemic arose from single/multiple spillover events from columbids to finches in GB and has been maintained predominantly by finch to finch transmission [30]. The frequency with which woodpigeon, *Columba palumbus*, and collared dove, *Streptopelia decaocto*, are reported from gardens has increased markedly since the 1980s, as did greenfinch; indeed, they were among the largest increases at garden feeding stations reported by Chamberlain *et al*. [8]. These increases are almost certainly due, in large part, simply to a larger population size; for example, woodpigeon numbers have increased in response to a greater area of oilseed rape cultivation [33]. It is also possible that a widespread change in the nature of garden feeding since the 1970s, with a large variety of food types provided year-round (increasingly presented in multiple feeders), has not only made gardens more attractive as foraging locations, but also increased the frequency of conspecific and interspecific interactions of birds at feeding stations.

Subsequent to its emergence in GB, finch trichomonosis spread to Fennoscandia in 2008 [34], with epidemiological and ring recovery data suggesting the chaffinch as the most likely primary vector [35]. Onward spread of finch trichomonosis has continued in mainland Europe, with molecular studies confirming the same clonal A1 strain of *T*. *gallinae* to be responsible [36].

### (b) Population-level impact

**Figure 1.**
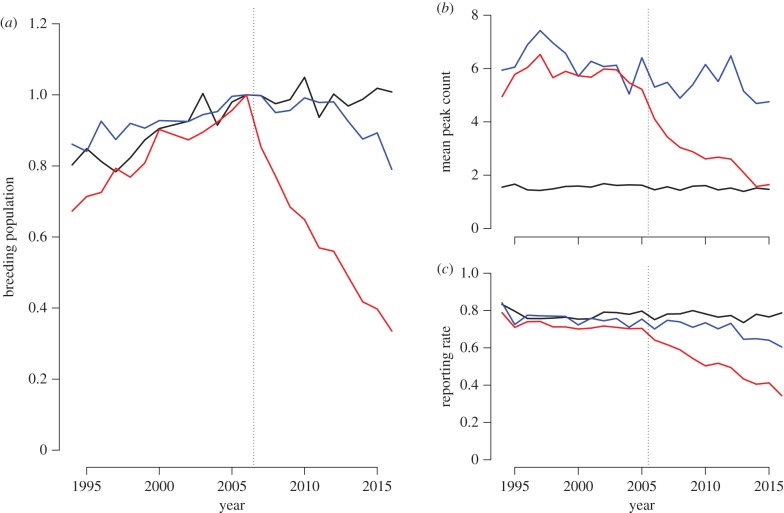
Number of greenfinch (red), chaffinch (blue) and dunnock (black). (*a*) Index of UK breeding population size from the BTO/JNCC/RSPB Breeding Bird Survey [37]; the index is set to 1 in 2006 representing a population size of 4.3 million (greenfinch), 14.3 million (chaffinch) and 4.4 million (dunnock) birds. (*b*) Mean maximum number of birds recorded each week in winter (October–March) in gardens at feeders from the BTO Garden Bird Feeding Survey. (*c*) Proportion of gardens in BTO Garden BirdWatch recording each species in winter. In all cases, the vertical dotted line indicates the timing of the initial finch trichomonosis epidemic in 2006.

In the first year of epidemic mortality (2006), a marked decrease in the number of gardens reporting greenfinches and chaffinches was noted, which was contemporaneous with a late summer peak in reports of the disease [29]. This resulted in a reduction in the breeding population of greenfinches (by 35%) and of chaffinches (by 20%) in the area with the highest frequency of disease reports, but no decrease in dunnock (*Prunella modularis*) numbers (which represents a rarely affected ‘control’ species [29]). In the years preceding the initial outbreak, all three species were widespread, being reported in approximately 80% of gardens each spring (figure 1*c*). In the years following the emergence of finch trichomonosis, dunnocks continued to be reported in a similar proportion of gardens to before, while the number of gardens reported to be visited by chaffinches decreased only slightly. Greenfinches, though, were seen in fewer than half (49%) the number of gardens in 2015/2016 relative to before the disease outbreak. At the same time, a large decrease in the number of greenfinches recorded at garden feeders was noted (from a mean of 5.6 average weekly maximum group size before 2006 to 1.6 in 2015/2016), with a smaller decrease for chaffinch (6.2 to 4.7) and no change was noted for the dunnock (mean = 1.5, figure 1*b*). These changes are reflected at the national level, as the size of the greenfinch (but not chaffinch or dunnock) breeding population has continued to decline markedly (by 66%, figure 1*a*), from a peak of approximately 4.3 million in 2006 (just before the onset of the epidemic) to approximately 1.5 million individuals in 2016 (i.e. an average reduction of approx. 280 000 individuals per annum). This represents the largest scale infectious disease impact on a European wild bird on record and has led to the inclusion of the British race of Greenfinch, *C. chloris harrisoni* on the red list of Birds of Conservation Concern [38]. Furthermore, recent assessment of extinction risk using the International Union for Conservation of Nature Red list guidelines classified the breeding greenfinch population in GB as endangered [39]. It is unclear whether the more recent decline in chaffinch numbers since 2012 (figure 1*a*) is a result of finch trichomonosis or other causes.

## 3. Paridae pox

Multiple strains of avian poxvirus are known to infect wild birds in GB, with house sparrow (*Passer domesticus*), starling (*Sturnus vulgaris*), woodpigeon and dunnock most commonly affected in garden habitats [28,40]. Avian pox most commonly presents as proliferative, and readily observable, skin lesions [41]. Sporadic reports of disease, mostly of mild to moderate severity, have been confirmed in GB for decades and usually affect individual or small numbers of birds only. Avian poxvirus is resistant in the environment and can persist in a viable state for long periods, estimated to be from weeks to months. Virus transmission occurs via a combination of routes, including direct and indirect contact, perhaps facilitated by skin breach and invertebrate vectors such as biting mosquitoes [41].

### (a) Pattern of occurrence of Paridae pox

In 2006, a novel and severe form of avian pox affecting Paridae species, most frequently the great tit (*Parus major*), was diagnosed in southeast England with multiple birds affected in the majority of reported incidents [42]. Reports were received throughout the year, but with a pronounced seasonal peak in the post-breeding period (August/September), when population densities are at their highest following recruitment of naive juveniles, but also coincident with a seasonal peak in the abundance of mosquito vectors. Following the initial cases, there was a marked expansion in the disease range, consistent with epidemic spread from a point source introduction [42].

Sequence analysis of the avian poxvirus strains affecting British garden bird species revealed that a single B1 clade virus was responsible for Paridae pox emergence, which was distinct from all other strains except that affecting dunnock, although these might be distinguished as separate strains at higher sequencing resolution [42]. This severe form of pox has been known at an apparently low prevalence in Scandinavia since the 1950s, with incidents seen elsewhere in mainland Europe since 2005 [43]. Analyses of the avipoxvirus 4b core protein gene showed identical DNA sequences to great tits in mainland Europe and GB [42,43]. The spatio-temporal pattern of spread and the same virus strain emerging in great tits in GB and mainland Europe support a hypothesis of disease emergence subsequent to virus incursion, rather than emergence in GB as a result of a spillover event, e.g. from dunnock. As great tits in GB are relatively sedentary, the introduction is likely to have occurred as a result of invertebrate vector movement, perhaps via wind-borne spread or accidental transport [42]. Great tits seem to be particularly susceptible to the disease because other tit species (such as the blue tit *Cyanistes caeruleus*) exposed to the same environment are affected much less frequently [44].

**Figure 2.**
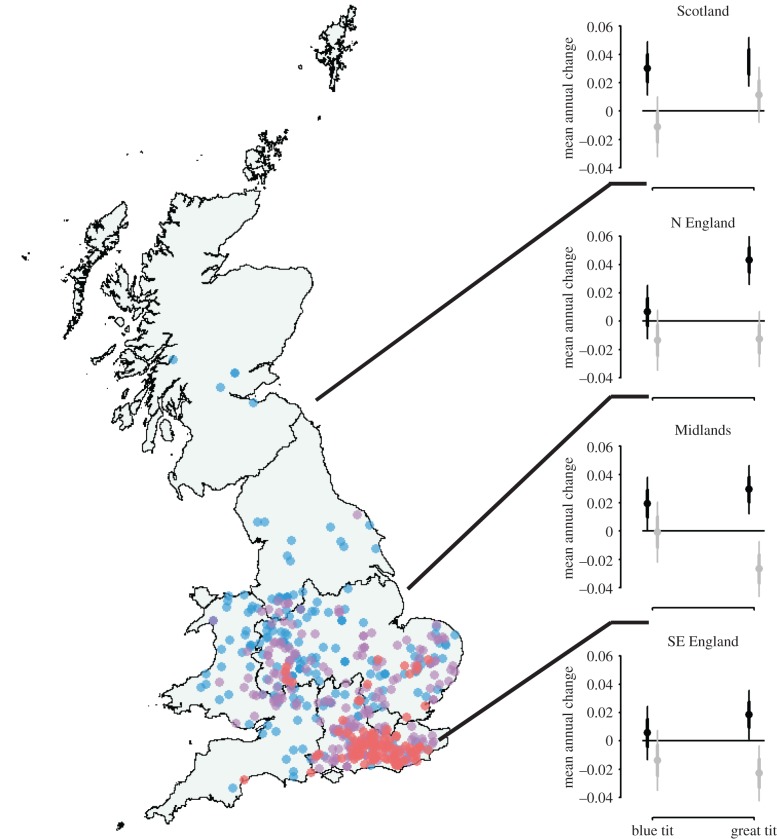
Records of Paridae pox in great tit submitted in 2006–2009 (red dots), 2010–2013 (purple) and 2014–2016 (blue). Graphs show population growth (mean annual change) for each delineated region for 1994–2005 (black, i.e. before Paridae pox emergence) and 2006–2016 (grey, i.e. after Paridae pox emergence). Points indicate the mean estimate, thicker bars indicate ±1 standard error and thin bars indicate 95% confidence limits. Regions follow the Government Office/NUTS boundaries.

Paridae pox incident reports, which have continued to be received (to 2016), demonstrate that the disease has persisted in southeast England and has an expanding range northward and westward across England and Wales; only a few reports have been received from Scotland since the first report from there in 2015 (figure 2).

### (b) Population impact

Although reports of Paridae pox have been received over a similar time period to those of finch trichomonosis, the extent of any regional- and national-scale population impacts are unclear, but appear to have been small. This difference may be, in part, because Paridae pox appears to have a comparatively lower mortality rate than finch trichomonosis and possibly also a lower rate of transmission. While finch trichomonosis rapidly spread across the whole country, and beyond [30], Paridae pox remained confined to the southeast of England for several years (2006–2009) following its initial emergence before reports started to be received from further afield [42]. In the 10 years prior to 2005, populations of great tits were increasing, but they have recently decreased significantly in southern England, but not in northern England, or Scotland where Paridae pox is still scarce (figure 2). While blue tit populations have also decreased since 2005, there was no difference between regions, in contrast to great tits (electronic supplementary material, table S1). These contrasting population trends are consistent with a disease-mediated decline of great tits in England, although the pattern of regional change in great tit population size does not match well with the expectation that population declines would be greatest in the southeast, where the disease has been present for the longest period. Thus, unlike with the greenfinch where finch trichomonosis is believed to be the major driver of population decline, factors other than infectious disease, such as winter weather, breeding season productivity or the frequency of tree masting, might be contributing to the changing population status of the great tit.

Capture–mark–recapture investigations of a population of great tits found a significant adverse effect of Paridae pox on fledging success and on host survival, especially in juveniles [45]. However, an age-structured population model accounting for these effects determined that while Paridae pox could cause a population-level decline, this was not predicted at the observed field prevalence of 5%, but would only occur if the disease prevalence exceeded 8%. There is thus a need for regional sampling to determine the levels of prevalence in great tit populations to inform predictions for future effects of this disease on great tit populations.

## 4. Passerine salmonellosis

*Salmonella enterica* serovar Typhimurium (definitive phage types (DT)160, 40 and 56 variant) causes salmonellosis in passerine birds and has been reported from various countries since at least the 1950s [46–50]. The disease is characterized by disseminated granulomatous lesions, most commonly affecting the oesophagus, liver, spleen and caecal tonsils. Transmission is faeco-oral and *Salmonella* bacteria are capable of persisting for many months within the environment. Incidents of passerine salmonellosis are typically reported in the vicinity of supplementary feeding stations [46–50]. Gregarious and granivorous species in the Fringillidae and Passeridae are most commonly affected by the disease, particularly the greenfinch and house sparrow in GB [48]. Passerine salmonellosis incidents have pronounced winter seasonality in GB, typically peaking in January. The wild bird species that are affected by disease are proposed as the reservoir of these *Salmonella* biotypes, which are considered to be host-adapted [51].

### (a) Pattern of occurrence of passerine salmonellosis

While the disease is considered to be endemic, with widespread occurrence, in GB, longitudinal studies have shown variation in the spatial distribution of the main phage types in England and Wales with succession of the predominant phage type over time, with shifts from DT160 to DT40 and then DT56(v) [52] (electronic supplementary material, figure S1*b*). In contrast to the patterns of occurrence of finch trichomonosis and Paridae pox, the number of confirmed passerine salmonellosis incidents has declined markedly over the past decade [52] (electronic supplementary material, figure S1*a*). The reason for the sharp decline in this endemic disease remains unknown, but we propose two potential hypotheses: first, that the decline in DT56(v) incidents reflects the development of herd immunity to this biotype and the populations are now vulnerable to emergence of a new variant; or, second, that passerine salmonellosis has density-dependent transmission, and the dramatic reduction in greenfinch numbers in garden habitats, due to finch trichomonosis, has had a secondary impact on occurrence of this bacterial disease. Serosurveys of wild bird populations may help elucidate these hypotheses.

### (b) Population impact

There have been anecdotal reports of temporary, localized reductions in bird numbers following passerine salmonellosis outbreaks [53]. There is, however, no evidence to indicate that passerine mortality caused by salmonellosis occurs at a scale sufficient to cause widespread wild bird population declines in GB.

## 5. Mycotoxin exposure

Exposure to aflatoxins (AFs) and ochratoxin A (OA) can exert a range of adverse effects in birds, ranging from acute toxicosis to chronic subclinical impairment of growth, reproduction and immune function [54]. Aflatoxins (B_1_, B_2_, G_1_ and G_2_) and OA are secondary metabolites produced by filamentous fungi of the genera *Aspergillus* and *Penicillium*, respectively, and have been shown to be produced in peanuts and other foodstuffs used in supplementary foods sold for garden bird consumption [55]. Optimal conditions for AF production occur at high temperature and relative humidity, although Thompson and Henke [56] demonstrated AF production under temperate climatic conditions; OA production also occurs in conditions found in GB [57]. Furthermore, hepatic AF residues have been detected in house sparrows and greenfinches found dead in gardens in GB, confirming exposure of wild birds in peri-domestic habitats in this country [58].

### (a) Pattern of occurrence of mycotoxin exposure

To determine whether garden feeding stations could act as a source of AF or OA in GB, a pilot study was conducted to screen for these mycotoxins in food residues collected from hanging feeders in use at feeding stations [59]. Food residues collected from hanging feeders were submitted from seven gardens in southeast England in 2005 (see the electronic supplementary material). Detectable AF residues were found in all seven samples, two of which greatly exceeded the 20 µg kg^−1^ maximum permitted limit for AFB_1_ (for peanuts in livestock feed, including wild bird food) [60] at values of 690 and 61 710 µg kg^−1^ (electronic supplementary material, table S2). Detectable OA residues were found in two samples (1.0 and 2.6 µg kg^−1^), neither of which approached the 100 µg kg^−1^ EU guidance limit for OA in poultry foodstuffs [61]. Thus, garden birds may be exposed to AF residues in supplementary food at levels associated with acute and chronic toxic effects in captive birds. Experimental studies have found variation between species in apparent ability to discriminate against AF-contaminated food sources, suggesting that avoidance may not be possible [62].

### (b) Population impact

Research on poultry species has shown marked interspecific variation in the effects of mycotoxins (e.g. [63,64]), but no information is currently available on the susceptibility of wild bird species. While AF residues were detected in the liver of common British garden birds, no evidence of macroscopic hepatic abnormalities was detected on post-mortem examination of these birds; microscopic appraisal of tissues was not possible [58]. The impacts of AF and OA exposure at both the individual and population level for wild birds are, therefore, currently unknown.

## 6. Identification of risk factors and future research needs

Provision of supplementary food, both intentional and unintentional, has occurred throughout human history and has shaped communities, food webs and ecosystems [65]. More specifically, provision of food at garden bird feeding stations has the potential to influence bird populations over a larger area, both directly through the energy and nutrients provided, but also through the alteration of pathogen dynamics, including the transmission of disease to a wider population [2]. It has been shown that the birds using garden feeding stations may be attracted from a much wider area, arriving to take advantage of supplementary food, especially when natural foods may be in short supply [8,23,66]. The finch trichomonosis work reviewed here highlights the potential importance of garden feeding stations in facilitating disease transmission that can adversely impact populations, as well as wild bird welfare. However, there is much that is still unknown about the risk factors associated with supplementary feeding.

There is a need to characterize basic epidemiological parameters (such as incubation periods, transmission rates and infection probability) and variation in species' susceptibility, although this would require experimental challenge studies for specific pathogens. Such information would enable parameterization of models to assess the relative benefits provided by increased food availability and the costs of disease transmission, but also to predict the effect of mitigation measures and their concomitant population-scale impacts. While peri-domestic habitats can support a substantial proportion of the overall population of some bird species [67], the extent of interchange of individuals between these and rural habitats is poorly known, and hence so is the extent to which diseases transmitted at feeders can infiltrate the wider population. Furthermore, reports of disease incidents observed in the vicinity of feeding stations may provide unrepresentative estimates of disease risk, because infected individuals frequently remain around feeding stations for long periods (e.g. [68]) and, therefore, are visible through to the end stage of disease [29]. Surveillance of species in rural locations away from established feeding stations is likely to be required to gain a more complete picture of the infection landscape. This approach was adopted in a study of multiple bird species at forested sites in central Illinois, USA, which contrasted individual bird health at sites with and without supplementary food provision [69]. Live bird capture in mist nets and sampling were employed with assessment of multiple parameters, appraising stress (i.e. heterophil-to-lymphocyte ratio), body condition (i.e. body fat score and body condition index), antioxidant capacity, total protein, haematocrit, feather quality, reproductive physiology (i.e. testosterone or oestradiol plasma concentration), immune function (i.e. microbial killing assays) and observation of clinical signs of disease (e.g. conjunctivitis, avian pox, fungal skin disease, cloacal infection). Both positive and negative impacts of wild bird feeding were detected. Results indicate that, in general, birds at sites with feeding stations were in better physiological condition and of greater overall health status; however, there was significantly greater observed disease prevalence than for birds at control sites. Similar studies in additional countries, with different wild bird species and infectious diseases, would be worthwhile and also could include pathogen screening and/or serosurveillance would be worthwhile.

A key unknown is exposure risk, both to pathogens and to toxins. While the impact of heavy metals and pesticides is often well studied (e.g. [70]), the role of mycotoxin contaminants in food supplies has largely been ignored. The occurrence of mycotoxin contamination is higher in warm, wet conditions, which are likely to become more frequent with climate change [71]. The ability of pathogens to persist in the environment is poorly known, yet this is a key determinant of exposure risk. For example, experiments have determined that *T. gallinae* can survive in moist seed (<24 h), seed with organic material (less than 48 h) and distilled water with organic material (less than 16 h), but no survival was detected in dry seed [72,73]. The method of food provision (e.g. ground, bird table or in a hanging feeder) will also influence the likelihood of exposure. For example, transmission of *T. gallinae* may be facilitated by horizontal feeding surfaces, where saliva and regurgitated food from infected birds can easily contaminate fresh food. By contrast, hanging feeders may increase direct and indirect contact rates via perches or mesh and facilitate avian poxvirus transmission. The presence of tube style feeders was found to be significantly associated with the risk of house finch conjunctivitis, a disease transmitted via direct and indirect contact [74]. Feeding may also lead to increased opportunities for interspecific mixing at close quarters, including of species that would not normally associate together, elevating the risk of pathogen transmission from one species to another. Observational studies of species using feeding stations, their community composition, behaviours, contact rates and dominance hierarchies may identify the species and/or individuals at greatest risk of pathogen exposure.

Provision of supplementary food can influence pathogen invasion and prevalence, and there is a need to explore interactions between host demography, contact behaviour and immune defence [75]. Empirical studies to assess the nutritional composition of supplementary food, and how this resource affects host immunity, would help inform the trade-off between risks and benefits. Poor-quality or contaminated food can compromise immune function, further increasing the risk of disease transmission [16]. Commercially available foods for garden birds are typically based on convenient, affordable and available seed resources, some of which are known to be nutritionally incomplete when they form the majority diet of captive birds (e.g. excess fat, deficient in vitamins A and D3 and calcium [76]). While provision of artificial food sources in garden habitats is proposed as a supplement to natural diets (when a balanced nutritional composition may not be required), there is a need for further investigation to determine the proportion of the diet that it constitutes [14]. While there have been a limited number of such studies with tit species which indicate that the proportion of supplementary food in the diet varies by site and individual (e.g. [77]), how this alters by species is unknown.

Evaluation of the risk of mycotoxin exposure associated with supplementary feeding could be conducted by comparing toxins in various provisioned food types to levels in natural wild foods. Measuring mycotoxin levels in provisioned food at the point of sale, after storage and following exposure to British climatic conditions would provide information about the risk of mycotoxin exposure, but this would also need a better understanding of how wild birds react to and consume foods with differing levels of mycotoxin. Identifying the impacts of mycotoxins on wild birds and how they vary by species would require studies involving experimental exposure to the toxins of interest.

While there is no known risk to public health from finch trichomonosis or Paridae pox, people can develop gastroenteritis following infection with garden bird-associated strains of *S.* Typhimurium. Evaluation of the spatio-temporal trends of the *S.* Typhimurium phage types seen in garden birds and matched biotypes in people over a 20-year period in England and Wales found evidence of a positive association in both time and space between the two cohorts [52]. Whole-genome sequence studies of *S*. Typhimurium phage types 40 and 56(v) from both garden birds and people in GB showed that they were genetically closely related [78]. While these combined data support wild birds as a potential source of zoonotic disease, it is important to note that wild bird-associated biotypes represented only 0.2% of human *Salmonella* infections over a 10-year period [52]. Risks to domesticated animal health from wild bird disease should similarly be considered.

Social science methodologies can be used to understand the motivations of people who feed garden birds [79] and the ways to most effectively communicate guidance to direct and effect improvements in practices for disease prevention and control. Parallel approaches being used to understand community participation in citizen science might be useful here [80]. The use of supplementary feeding to support recovery programmes for species of conservation concern has generated a strategic and integrated approach, applying structured decision-making processes to the critical evaluation of benefits and risks for individual conservation projects [81]. A similar approach may be tenable on a larger scale to facilitate the development of best-practice guidance for feeding garden birds integrating available information from various disciplines.

## 7. Evidence-based mitigation strategies to address anthropogenically mediated pathogen transmission

While there remain many unanswered questions, there is a need to offer strategic recommendations for disease prevention and control [82,83]. This should be based on our current understanding and apply the precautionary principle that accepts that supplementary feeding is likely to contribute to the transmission of certain infectious diseases [16].

### (a) Disease prevention

Guidance for disease prevention can be tailored to address potential risk factors; for example, offering a variety of supplementary foods from accredited sources and feeding in moderation or only providing an amount of food at a time that will be consumed within a short period (1–2 days). Perhaps most challenging in this regard is the identification of ways to provide supplementary food while minimizing opportunities for interspecific contact that might not regularly occur in the wild. Currently, there is a move towards providing a variety of food types in close proximity, which is likely to increase opportunities for species mixing: for example, on single pole multipurpose feeding platforms that accommodate suspended feeders for seed and nuts, together with platform feeders and water bowls. Measures to reduce contamination at feeding stations, including regular cleaning and disinfection of bird feeders and tables, removal of food waste and faeces, and frequent replenishment and rotation of the locations of feeding stations are recommended. Feeder designs could be improved to reduce the chances of deterioration in food quality (e.g. prevent food becoming moist), avoid contamination of uneaten food and promote ease of cleaning. Food should also be stored appropriately and kept dry, with no rodent access.

As wildlife diseases are often highly seasonal, avoiding feeding during sensitive periods has been suggested as a measure to mitigate spread of diseases associated with population-level declines, such as during the house finch conjunctivitis peak in the autumn months [16]. However, a study using large-scale surveys found that house finch declines following disease emergence were greatest where the density of bird feeding was reduced, which indicates that while supplementary food provision may contribute to pathogen transmission, there might have been some compensatory positive effect countering the level of disease-related mortality [84]. Comparison of these findings with additional studies, focused on alternative infectious diseases with varying case fatality rates, would be useful to further appraise the evidence for beneficial effects of feeding versus detrimental effects of increased transmission opportunities. The potential benefit of food withdrawal to reduce opportunities for pathogen spillover to other wild bird species, which is a particular concern for the transmission of finch trichomonosis to other passerine species, requires similar evaluation.

It is worth noting that compliance with any request to cease feeding for disease prevention is likely to be ignored by a significant proportion of those people who feed wild birds. Motivations to feed are varied, including pleasure, environmental and philosophical preconceptions about wild bird care and responsibility [14]. This suggests that efforts could more effectively be directed to moderating behaviour in other ways, perhaps by seeking to reduce or redistribute the volume or type of food resources made available, or by pursuing the adoption of optimal hygiene measures. Educating the general public about the typical signs of ill health in garden birds, raising awareness of the occurrence and impact of disease outbreaks and communicating the benefits of vigilance for early detection and diagnosis may all assist with public understanding and the acceptance of evidence-based, best-practice advice. Research into the public's perception of who is responsible for investigation and action during wildlife disease outbreaks and their opinions on available mitigation measures would be useful.

### (b) Disease control

Control measures applicable to disease outbreaks in garden birds are limited. While requests from the public to medicate free-living birds are frequently received, this practice is not recommended for multiple reasons, including the inability to safely and effectively provide the correct dose to target free-living animals and the risk of promoting antimicrobial resistance. Treatment is only practicable if affected garden birds are taken into care; however, because small passerines usually can only be captured by hand at the end stage of disease, these casualties typically have a poor prognosis [85]. Where multiple birds are affected during a disease outbreak, temporary suspension of feeding to disperse birds, to reduce local population density and to reduce the risks of intra- and interspecific spread, is often advised. This recommendation may be influenced by the mode of transmission of the disease and how likely it is that the rate of transmission will be influenced by congregation at feeding stations. Suspension of bird feeding is also advocated where there is suspicion or confirmation of potentially zoonotic disease, such as passerine salmonellosis, to reduce the risk of public exposure through continuation of the activity.

## 8. Conclusion

This review summarizes field data on a national scale that clearly demonstrates the dynamism of endemic and emerging diseases in wild bird populations, even within a relatively small geographical area, such as GB, and a short time frame. The aetiological agents, mechanisms of emergence, modes of transmission and anthropogenic activities likely to influence transmission vary. In a world where the focus is typically on emerging conditions (e.g. finch trichomonosis and Paridae pox), the marked reduction of an endemic disease (e.g. passerine salmonellosis) reinforces that changes in occurrence can be bidirectional and that there may be interplay between the conditions present. Our findings highlight the importance of longitudinal scanning surveillance to capture early signals of changes in disease epidemiology, not just the emergence of novel conditions. Such surveillance informs the real-time prioritization of recommendations for mitigation tailored to current conditions and their concomitant risks to both wild bird and public health. There is a need to balance the risks and benefits of supplementary feeding of garden birds to both wildlife and people, which can be facilitated by engaging with the general public and the bird food industry to promote understanding and to encourage compliance with best-practice guidance.

## Supplementary Material

Supplementary Materials

## Data accessibility

Review article, see cited publications. The mycotoxin analyses results on bird feeder residues are available in electronic supplementary material, table S1.
