# Supplementary material for: Health hazards to wild birds and risk factors associated with anthropogenic food provisioning
Source: Philos Trans R Soc Lond B Biol Sci. 2018 Mar 12;373(1745):20170091. doi: 10.1098/rstb.2017.0091 (PMC5882997; doi:10.1098/rstb.2017.0091)
Supplement: Supplementary Materials [file rstb20170091supp1.docx]

**Health hazards to wild birds and risk factors associated with anthropogenic food provisioning**

**Becki Lawson 1, Robert A. Robinson^2^, Mike P. Toms^2^, Kate Risely^2^, Susan MacDonald^3^, Andrew A. Cunningham^1^**

*^1^Institute of Zoology, Zoological Society of London, Regent’s Park, London, NW1 4RY, UK*

*^2^British Trust for Ornithology, The Nunnery, Thetford, Norfolk, IP24 2PU, UK*

*^3^Fera Science Ltd, National Agri-Food Innovation Campus, Sand Hutton, York, YO41 1LZ, UK*

**Supplementary Materials**

**Supplementary Methods**

1. **Post-mortem examination protocol**

Post-mortem examinations were conducted following a standardised protocol [1 - 2]. During the period 2005-2010, a subset of the examinations was performed at a network of regional veterinary diagnostic laboratories. In total, circa 4000 PMEs of over 60 wild bird species were conducted from 1992-2016 inclusive.

Details of the species, age, sex, body weight and a subjective assessment of body condition (based on body fat and muscle condition) were recorded for each bird examined. Systematic external and internal examinations of body systems were performed and any macroscopic lesions described. Where indicated, and where the state of carcass decomposition permitted, samples were taken for microbiological, parasitological and histopathological investigations.

Liver and contents of the mid-small intestinal loop were sampled from the majority of cases, as were any macroscopic lesions detected, and were examined for the presence of pathogenic bacteria using a standardised protocol including Salmonella-selective enrichment media [2]. Bacterial isolates were identified using colony morphology and Gram's staining coupled with biochemical properties, which were determined using the analytical profile index (API) 20 Enterobacteriaceae biochemical test strip method (API-BioMerieux, Marcy l'Etoile, France). Slide agglutination tests were performed for the identification of suspected Salmonella spp. isolates using poly-O antisera (Pro-lab diagnostics, Neston, UK).  Biotyping (serotype and phage type) of *Salmonella* sp. isolates was conducted according to standardised international protocols [3].

Oesophageal lesions (circa 5 mm^3^) from cases with necrotic ingluvitis were incubated at 30°C in Trichomonas Media No. 2. (Oxoid, UK) and screened for motile trichomonads at 24, 48, 72 hrs and 5 days [1]. Wet mount preparations of small intestinal contents were examined in a subset of cases for evidence of metazoan and protozoan parasites.

Samples from a range of organs and lesions, including suspect avian pox skin lesions when present, were fixed in neutral-buffered 10% formalin and processed for histopathological examination using routine methods. Avian pox was confirmed on histological observation of intracytoplasmic eosinophilic inclusions (Bollinger bodies), considered pathognomonic for the disease [4].

Duplicate samples of organs and macroscopic lesions were archived at −20°C or −80°C. DNA extraction from macroscopic lesion and PCR amplification was used in some birds to confirm diagnoses of finch trichomonosis and avian pox when suspected, targeting the trichomonad ITS1/5.8S/ITS2 ribosomal region or trichomonad small subunit rRNA [1] and core 4b protein gene of avipoxvirus respectively [4].

1. **Standardised case and incident definitions**

Definitions employed in this study, include:

1. Finch trichomonosis case definition: confirmed diagnoses where finches had macroscopic lesions of necrotic ingluvitis in combination with positive culture of motile trichomonads and/or positive PCR amplification of *Trichomonas gallinae*. Suspected diagnoses where finches had macroscopic lesions of necrotic ingluvitis, and were negative for *Salmonella* sp. isolation, but *T. gallinae* not confirmed by parasite isolation and PCR was not performed [1].
2. Paridae pox incident definition: sites where one or more tits (great tit and/or blue tit and/or coal tit) were observed with descriptions corresponding to proliferative skin lesions on head or body (e.g. “growths”, “swellings”, “lumps”, “tumours”): photographs of affected birds when available were reviewed by a veterinary surgeon to confirm that the macroscopic appearance was typical of the disease [4].
3. Passerine salmonellosis incident definition: sites where one or more birds had a *Salmonella* sp. isolated from a macroscopic lesion, or lesions, characteristic for salmonellosis in the absence of any other obvious cause of death [5].

**(c ) Bird feeder residues collection and mycotoxin analyses**

Participants emptied the food contents from the bottom of their feeders in use in their gardens when they were almost empty into a sterile universal tube and also scraped any residues into the container. Samples were submitted within 48 hours of collection and stored at -20 °C prior to testing. For each sample, the location, date of sampling, food type, and estimated duration of time that the food had been within the feeder (i.e. period since the feeder was last cleaned) was recorded.

Bird feeder residues were weighed and a homogenised slurry was created from each sample by adding 200 ml of tap water per 250 g of food product (peanuts or mixed seed) and grinding the sample in a food processor for 20 minutes. Analysis of mycotoxins was carried out using fully validated and accredited (to ISO 17025 by the United Kingdom Accreditation Scheme) methods based on those published for aflatoxins(AF) [6] and ochratoxin A (OA) [7] or a combined AF/OA method [8] depending on the availability of clean-up columns: validation and quality control procedures have shown that single or combined methods produce equivalent results [8]. For all analyses, the slurried sample was extracted with acetonitrile / deionised water and the extracts were filtered, diluted and cleaned up by immunoaffinity column.

Clean-up and reverse phase high performance liquid chromatography (HPLC) with fluorescence detection (with post column derivatisation for aflatoxins B_1_ and G_1_) were carried out using a fully automated system [6 - 8]. Normal in-house quality control, including the use of in-house reference materials, blank and spiked matrix samples were used in each batch run to ensure that variation between batches remained within acceptable limits. For very high concentration samples, adjustments were made, by diluting samples, to ensure clean-up columns were not overloaded, and that the samples analysed were within the calibration range. The concentrations (µg/kg) of mycotoxins detected were corrected according to the percentage of mycotoxin recovery achieved in each batch analysed, as determined from the results for the spiked samples.

**Supplementary Figure 1**

1. Number of garden bird mortality incidents diagnosed with garden bird-associated Salmonella Typhimurium phage types, 1992-2016 (b) Percentage breakdown of garden bird-associated Salmonella Typhimurium phage types, 1992-2016

*Salmonella* Typhimurium phage type (DT)40 (red), DT56(v) (blue) and DT160 (green). See Supplementary Methods for passerine salmonellosis incident definition. Figure combines available data from England and Wales by IoZ and the Animal & Plant Health Agency. Whilst the intensity of surveillance varied over the period 1992-2004, a comparable surveillance effort and consistent methodology has been used for garden birds in England & Wales from 2005-2016 inclusive. Figure (a) updated and modified from figure 2 in [2]

(a)

(b)

**Supplementary Table 1**: Regional trends in blue and great tit populations.

Models fitted included a simple linear year (Y) term; a linear year term separately for the years 1995-2005 (1) and 2006-2016 (2), representing the pre and post Paridae pox periods (Y:P); a single year term for each region (R:Y) or a separate year term for each region and period (R:Y:P); all models included a regional intercept (separately by period if appropriate). The table gives the parameter estimates ± 1 standard error and model AIC, with the lowest AIC model highlighted. Regions followed European NUTS boundaries, with the Midlands region combining East/West Midland and Eastern England, and North England combining North-east/west England and Yorkshire and the Humber.

|  | **Blue Tit** | | | | |  | **Great Tit** | | | | |
| --- | --- | --- | --- | --- | --- | --- | --- | --- | --- | --- | --- |
| **Model** | **SE England** | **Midlands** | **North England** | **Scotland** | **AIC** |  | **SE England** | **Midlands** | **North England** | **Scotland** | **AIC** |
| Y | 0.001 ± 0.002 | | | | -114.6 |  | 0.023 ± 0.002 | | | | -76.2 |
| Y:P (1)  (2) | **0.015 ± 0.005**  **-0.025 ± 0.007** | | | | **-122.4** |  | 0.031 ± 0.005  -0.044 ± 0.007 | | | | -129.3 |
| R:Y | -0.003 ± 0.007 | 0.011 ± 0.008 | -0.002 ± 0.008 | 0.012 ± 0.008 | -109.4 |  | 0.001 ± 0.008 | 0.005 ± 0.008 | 0.019 ± 0.008 | 0.024 ± 0.008 | -94.8 |
| R:Y:P (1)  (2) | 0.006 ± 0.010  -0.014 ± 0.011 | 0.019 ± 0.010  -0.001 ± 0.011 | 0.007 ± 0.010  -0.013 ± 0.011 | 0.030 ±0.010  -0.011 ± 0.011 | -116.8 |  | **0.019 ± 0.009**  **-0.022 ± 0.010** | **0.029 ± 0.009**  **-0.027 ± 0.010** | **0.043 ± 0.009**  **-0.012 ± 0.010** | **0.035 ±0.009**  **0.012 ± 0.010** | **-132.1** |

**Supplementary Table 2**: Aflatoxin and Ochratoxin A levels in Bird Feeder Residues (μg/kg)

For comparative purposes, maximum permitted limits for AFB_1_ (for peanuts in livestock feed, including wild bird food) in England are 20 μg/kg [9] and the EU guidance limit for OA in poultry foodstuffs is 100 μg/kg [10].

| Sample | AFB_1_ | AFB_2_ | AFG_1_ | AFG_2_ | Total AF | OA | Feeder type | Food type | Estimated time in feeder |
| --- | --- | --- | --- | --- | --- | --- | --- | --- | --- |
| 1 | 0.9 | <0.2 | <0.2 | <0.2 | 0.9 | <0.2 | Wire mesh | Unbranded seed & unbranded peanuts | 5 months |
| 2 | 1.7 | 1.5 | <0.2 | <0.2 | 3.2 | <0.2 | Wire mesh | Branded peanuts | >1 year |
| 3 | 0.7 | <0.2 | <0.2 | <0.2 | 0.7 | <0.2 | Plastic seed hopper | Branded seed | 3 months |
| 4 | 7.4 | 1.5 | 6.1 | 1.5 | 16.5 | 1.0 | Wire mesh & Plastic seed hopper | Branded seed & Unbranded peanuts | 3 months |
| 5 | 61,710 | 4,810 | 39,850 | 2,730 | 109,100 | <0.2 | Wire mesh | Branded peanuts | 3 weeks |
| 6 | 690 | 28.2 | 181 | 11.2 | 910 | <0.2 | Plastic seed hopper | Non branded seed | 4 months |
| 7 | 4.2 | 0.5 | 1.1 | <0.2 | 5.8 | 2.6 | Wire mesh | Branded peanuts | 6 months |

**References**

1 Robinson, R.A., Lawson, B., Toms, M.P., Peck, K.M., Kirkwood, J.K., Chantrey, J.,  Clatworthy, I.R.,  Evans, A.D., Hughes, L.A., Hutchinson, O.C., et al. 2010 Emerging infectious disease leads to rapid population declines of common British birds. PLoS ONE **5(8)**, e12215. ([doi:10.1371/journal.pone.0012215](http://journals.plos.org/plosone/article?id=10.1371/journal.pone.0012215))

2 Lawson, B., de Pinna, E., Horton, R.A., Macgregor, S.K., John, S.K., Chantrey, J., Duff, J.P., Kirkwood, J.K. , Simpson, V.R., Robinson, R.A., et al. 2014 Epidemiological evidence that garden birds are a source of human salmonellosis in England and Wales. *PLoS ONE* **9(2),** e88968. (doi:10.1371/journal.pone.0088968)

3 Anderson, E.S., Ward, L.R., de Saxe, M.J. & De Sa, J.D.H. 1977 Bacteriophage-typing designations of Salmonella typhimurium. *J. Hyg*. **78**, 297–300.

4 Lawson, B., Lachish, S., Colvile, K.M., Durrant, C., Peck, K.M., Toms, M.P., Sheldon, B.C. & Cunningham, A.A. 2012 Emergence of a novel avian pox disease in British tit species. PLoS ONE **7(11)**, e40176. ([doi:10.1371/journal.pone.0040176](http://dx.plos.org/10.1371/journal.pone.0040176))

5 Lawson, B., Howard, T., Kirkwood, J.K., Macgregor, S.K., Perkins, M., Robinson, R.A., Ward, L.W. & Cunningham, A.A. 2010 The epidemiology of salmonellosis in garden birds in England and Wales, 1993 to 2003. Ecohealth **7(3),** 294-306. (doi: 10.1007/s10393-010-0349-3)

6 Sharman, M. & Gilbert. J 1991 Automated aflatoxin analysis of foods and animal feeds using immunoaffinity column clean-up and high-performance liquid chromatographic determination. *J Chromatogr.* **543**, 220-225.

7 Sharman, M., MacDonald, S. & Gilbert. J 1992 Automated liquid chromatographic determination of ochratoxin A in cereals and animal products using immunoaffinity column clean-up. *J Chromatogr.* **603**, 285-289.

8 Chan, D., MacDonald, S.J., Boughtflower, V. & Brereton, P. 2004 Simultaneous determination of aflatoxins and ochratoxin A in food using a fully automated immunoaffinity column clean-up and liquid chromatography-fluorescence detection. *J Chromatogr.* **1059**, 13-16.

9 Anon. 2004 The Feeding Stuffs, the Feeding Stuffs (Sampling and Analysis) and the Feeding Stuffs (Enforcement) (Amendment) (England) (No.2) Regulations 2004 Statutory Instrument No. 2688. HMSO, 2004, pp. 1-12.

10 Anon. 2006 Council Recommendation 2006/576/EC of 17 August 2006 on the on the presence of deoxynivalenol, zearalenone, ochratoxin A, T-2 and HT-2 and fumonisins in products intended for animal feeding. Official Journal of the European Communities, L 229/7, 23/08/2006, pp. 1-3.
